# Involvement of Various Enzymes in the Physiology and Pathogenesis of *Streptococcus suis*

**DOI:** 10.3390/vetsci7040143

**Published:** 2020-09-23

**Authors:** Chengkun Zheng, Man Wei, Mengdie Jia, ManMan Cao

**Affiliations:** 1 Joint International Research Laboratory of Agriculture and Agri-Product Safety, The Ministry of Education of China, Yangzhou University, Yangzhou 225009, China; fhzxmwei@163.com (M.W.); jiamengdie@163.com (M.J.); 2 Jiangsu Key Laboratory of Zoonosis, Yangzhou University, Yangzhou 225009, China; 3 Guangdong Maoming Agriculture & Forestry Techical College, Maoming 525000, China; cmm1506225266@163.com

**Keywords:** *Streptococcus suis*, enzymes, physiology, pathogenesis

## Abstract

*Streptococcus suis* causes severe infections in both swine and humans, making it a serious threat to the swine industry and public health. Insight into the physiology and pathogenesis of *S. suis* undoubtedly contributes to the control of its infection. During the infection process, a wide variety of virulence factors enable *S. suis* to colonize, invade, and spread in the host, thus causing localized infections and/or systemic diseases. Enzymes catalyze almost all aspects of metabolism in living organisms. Numerous enzymes have been characterized in extensive detail in *S. suis*, and have shown to be involved in the pathogenesis and/or physiology of this pathogen. In this review, we describe the progress in the study of some representative enzymes in *S. suis*, such as ATPases, immunoglobulin-degrading enzymes, and eukaryote-like serine/threonine kinase and phosphatase, and we highlight the important role of various enzymes in the physiology and pathogenesis of this pathogen. The controversies about the current understanding of certain enzymes are also discussed here. Additionally, we provide suggestions about future directions in the study of enzymes in *S. suis*.

## 1. Introduction

*Streptococcus suis* is an important bacterial pathogen that causes a severe threat to public health and great economic losses in the pig industry worldwide [1]. It is associated with meningitis, septicaemia, arthritis, and other infections in swine [2]. A recent survey revealed that it was the most prevalent bacterial pathogen in Chinese pig farms from 2013 to 2017 [3]. In addition, *S. suis* can be transmitted to humans and cause severe infections, such as meningitis, septicemia, and streptococcal toxic shock-like syndrome [1]. *S. suis* infection in human was first recorded in Denmark in 1968 [2]. Since then, human cases have been reported in many countries, although most of them are sporadic [4]. However, two large outbreaks of *S. suis* epidemics occurred in China in 1998 and 2005, resulting in 240 human cases with 53 deaths in total [5,6]. The repeated outbreaks of *S. suis* infections in humans have suggested that *S. suis* is an emerging zoonotic pathogen [2]. By the end of 2013, worldwide reported *S. suis* infections in humans reached 1642 cases [1]. Human cases have also been frequently reported worldwide in recent years [7,8,9,10,11], suggesting that the threat of *S. suis* infection still exists.

*S. suis* possesses capsular polysaccharides (CPS) that cover on the surface of bacterial cells [12]. Initially, 35 serotypes (1–34, and 1/2) were described for *S. suis*, based on the serological reaction with its CPS [13]. Subsequently, serotypes 20, 22, 26, and 32–34 were proposed to be novel bacterial species, and the remaining 29 serotypes were referred to as genuine *S. suis* [14,15,16,17]. Globally, serotype 2 (*S. suis* 2) is the most predominant serotype associated with clinical *S. suis* infection in both swine and humans [1].

In swine, the main route of *S. suis* infection is the upper respiratory tract, yet the gastrointestinal tract cannot be excluded as a secondary route. In China and in Western countries, small skin injuries are considered to be the main route of *S. suis* infection in humans, while in Southeast Asian countries, the gastrointestinal tract (consumption of contaminated pork by-products) seems to be the main route [12,18].

During the infection process, a wide variety of virulence-associated factors enable *S. suis* to colonize, invade, and spread in the host, thus causing localized infections and/or systemic diseases [12,19]. Enzymes function as catalysts that catalyze almost all aspects of metabolism in living organisms. It has been well established that many enzymes are involved in the pathogenesis of *S. suis* [19]. Moreover, some enzymes play important roles in bacterial physiology, even though they are not virulence-associated factors. In this review, we emphasize the role of some representative enzymes in the physiology and pathogenesis of *S. suis* (Table 1), and we also discuss the controversies about the current understanding of certain enzymes.

## 2. ATPases

Recently, we identified two P-type ATPases (CopA and PmtA) that function as metal efflux pumps in *S. suis* [20,21]. In certain Gram-positive bacteria, *copA* is a component of the *cop* operon that is a copper-responsive system [67,68]. Although not arranged as an operon in *S. suis*, *copA* expression is significantly upregulated when the bacterium is treated with copper. In line with this result, CopA protects *S. suis* against bactericidal effects conferred by copper through copper efflux [20]. While *pmtA* expression is induced by ferrous iron, cobalt, and nickel, the Δ*pmtA* mutant exhibits impaired growth under ferrous iron, ferric iron, cobalt, and zinc excess conditions. Compared with the wild-type (WT) and complementation strains, Δ*pmtA* also accumulates higher levels of iron and cobalt, and is more sensitive to streptonigrin, a ferrous iron-activated antibiotic. The addition of manganese could alleviate the growth defect of Δ*pmtA* under ferrous iron and cobalt excess conditions. Furthermore, PmtA is involved in the tolerance to oxidative stress induced by hydrogen peroxide [21]. Despite the homologs of CopA and PmtA having been shown to be required for bacterial virulence [69,70,71,72], these two ATPases have exhibited no obvious role in the pathogenesis of *S. suis* [21,44,45]. Thus, it is necessary to study even well-characterized genes in different species.

In addition to CopA and PmtA, another ATPase, MsmK, has been identified in *S. suis* [22,23]. Similar to its homologs in *Streptococcus pneumoniae* [73], MsmK is required for the utilization of multiple carbohydrates, such as raffinose, melibiose, and maltotetraose in *S. suis* [22]. Deletion of *msmK* results in a longer chain length, decreased hemolytic activity, and an impaired ability to tolerate osmotic and oxidative stresses [23]. The Δ*msmK* mutant also exhibited reduced survival in mouse blood, increased susceptibility to macrophages, and an attenuated ability to colonize the mouse brain [22,23]. These results clearly demonstrated that MsmK contributes to the pathogenesis of *S. suis*.

## 3. Immunoglobulin-Degrading Enzymes

Immunoglobulins (Igs) are important components of the host immune defense system. Based on the differences in their heavy chains, Igs are divided into five classes, including IgG, IgA, IgM, IgD, and IgE. In *S. suis*, two Ig-degrading enzymes, i.e., Ide_Ssuis_ and IgdE, have been extensively characterized [24,25,26,27,74]. Ide_Ssuis_ is a novel IgM-degrading enzyme that can degrade IgM but neither IgG nor IgA. Ide_Ssuis_ is host-specific, since it specifically cleaves porcine IgM, but not IgM from six other investigated species [24]. Vaccination of piglets with recombinant Ide_Ssuis_ elicited specific immunity that led to the efficient killing of *S. suis* in porcine blood, and thus, protected piglets against *S. suis* infection [74]. In addition, IgM cleavage activity of Ide_Ssuis_ is involved in complement evasion, although it does not seem to be critical for the virulence of *S. suis* in piglets [25,26]. IgdE is a novel IgG-degrading protease that targets the hinge region of porcine IgG. Similar to Ide_Ssuis_, only porcine IgG can be a substrate of IgdE. Therefore, this enzyme is also host-specific. The IgG proteolytic activity is present in all *S. suis* strains investigated, and specific antibodies against IgdE were detectable in piglet serum [27]. These findings suggest that IgdE is expressed during infection; thus, it is a putative virulence factor and a potential vaccine candidate.

Another Ig-degrading enzyme, i.e., IgA1 protease, has been reported to cleave human IgA1 [28]. This enzyme has also been shown to contribute to the pathogenesis of *S. suis* and to serve as a protective antigen [29,75]. However, IgA1 protease activity was not detected in three *S. suis* strains in another study [30]. Recently, the enzyme was demonstrated to be ZmpC, a zinc metalloprotease in *S. suis*. Moreover, ZmpC is not a critical virulence factor, as it has no role in adherence to porcine bronchial epithelial cells or colonization of the upper respiratory tract of pigs [31].

## 4. Eukaryote-Like Serine/Threonine Kinase and Phosphatase

The eukaryote-like serine/threonine kinases and phosphatases (eSTKs/eSTPs) have important roles in the physiology and pathogenesis of *Streptococci* [76]. In *S. suis*, eSTK is involved in bacterial morphology, stress tolerance, and pathogenesis [32,33,34]. The eSTK-deletion mutant exhibits much longer chain length and increased cell size [32,33]. Subsequent research revealed that DivIVA, a substrate of eSTK, is involved in cell division regulation [77]. The morphological differences between the WT strain and the mutant might be due to the different phosphorylation levels of DivIVA in these two strains. The mutant displayed impaired growth when cultured under stress conditions, including high temperature, high osmolarity, acidic pH, and oxidative stresses [32]. In line with these phenotypes, seven metabolic pathways were significantly repressed in the mutant [33]. The involvement of eSTK in *S. suis* pathogenesis has been demonstrated by the following findings: firstly, the mutant showed a decreased ability to adhere to and invade cells, and had reduced survival in pig whole blood [32,34]; secondly, the ability of the mutant to cross the blood-brain barrier (BBB) was reduced [34]; thirdly, virulence of the mutant was attenuated in both murine and pig infection models; finally, the expression of some virulence-associated genes were down-regulated in the mutant [32,33]. These findings suggest that in *S. suis*, eSTK participates in multiple steps of the infection process.

eSTP has been identified as a putative virulence factor of *S. suis* serotype 9 by suppressing subtractive hybridization. Furthermore, the eSTP gene was present in most of the virulent strains, but absent in the avirulent strain. In *S. suis* 9, the eSTP-defective mutant exhibited decreased adherence to HEp-2 cells, reduced survival in pig whole blood, and attenuated virulence in the murine infection model. Consistent with these results, the expression of a few genes involved in adhesion and virulence was down-regulated in the mutant [35]. As has been observed in *S. suis* 9, deletion of eSTP attenuated the virulence of *S. suis* 2 in a murine infection model. However, the eSTP mutant of *S. suis* 2 displayed an enhanced ability to adhere to HEp-2 and bEnd.3 cells, to survive in RAW 264.7 macrophage cells, and to resist reactive oxygen species. The role of eSTP in cell adhesion and immune evasion seems to be contradictory between *S. suis* 2 and 9. Given that eSTP shares high level of homology between *S. suis* 2 and 9 [36], further studies should be performed to determine whether the opposite conclusions are attributable to the different serotypes.

## 5. Subtilisin-Like Serine Proteases

The subtilisin-like serine protease-1 (SspA-1) was identified by screening a *S. suis* 2 genomic expression library using convalescent-phase pig sera [37]. Later, SspA-1 was identified as an effector secreted by the type IV secretion system (T4SS) of *S. suis* 2 by using a shotgun proteomics approach and western blot analysis [38]. Quantitative real-time PCR analysis demonstrated that SspA-1 expression in vivo was markedly higher than that *in vitro*, indicating that SspA-1 might be involved in *S. suis* virulence [37]. Consistent with this speculation, the SspA-1 knockout mutant exhibited attenuated virulence in both murine and pig infection models [37,38]. Compared with the WT strain, the mutant induced much lower levels of interleukin 6, tumor necrosis factor-α, and interleukin 12p70 in mice; treatment of THP-1 cells with purified recombinant SspA-1 resulted in massive production of these cytokines. These results suggested that SspA-1 plays an important role as a trigger of proinflammatory cytokines. Furthermore, the reaction of SspA-1 with convalescent-phase pig sera revealed that SspA-1 might be a protective antigen. As expected, immunization of mice with SspA-1 elicited a specific immune response, producing a SspA-1 specific antibody that protected mice against *S. suis* infection [38].

The subtilisin-like serine protease-2 (SspA-2) was identified by screening a *S. suis* mutant library to isolate mutants deficient in proteinase activity. The *sspA-2* gene was present in all detected *S. suis* strains, including serotype 2 and other serotypes [39]. Different from SspA-1, the secretion of SspA-2 was not affected by T4SS [38]. A contribution of SspA-2 to the pathogenesis of *S. suis* has been revealed by several lines of evidence. Firstly, the recombinant SspA-2 displayed toxicity against brain microvascular endothelial cells. Secondly, SspA-2 could react with convalescent-phase pig sera, suggesting that it is expressed during infection [40]. Thirdly, the SspA-2 inactive mutant was more susceptible to killing by human whole blood. Fourthly, mice infected with the mutant had a lower mortality rate than those infected with the WT strain [39]. Finally, SspA-2 induced a pro-inflammatory response in macrophages, which might promote meningitis [41].

## 6. Superoxide Dismutase and NADH Oxidase

Superoxide dismutase (SOD), usually coupled with a metal cofactor, can catalyze the reaction of superoxide to oxygen and hydrogen peroxide, and it is involved in oxidative stress resistance and virulence in many bacterial species [78]. In *S. suis*, manganese, instead of iron, is required for the activity of SOD [79]. The *sod* gene deletion mutant loses its SOD activity, and it is more sensitive to hydrogen peroxide and paraquat-induced oxidative stress. The mutant also shows decreased survival in RAW264.7 macrophages, attenuated virulence in mice, and an impaired ability to colonize the tissues of mice [42]. Subsequently, further studies demonstrated that the involvement of SOD in anti-autophagic responses was mediated by the scavenging of reactive oxygen species (ROS) in infected macrophages [43]. Superoxide dismutase appeared to be regulated by the two-component system Ihk/Irr and the SpxA1 regulator in *S. suis*, since expression of the *sod* gene was significantly down-regulated in the Ihk/Irr and *spxA1* deletion mutants [80,81].

NADH oxidase (Nox) can catalyze the reduction of oxygen to hydrogen peroxide or water, combined with the oxidation of NADH to NAD^+^ in bacteria [82]. In *Streptococcus mutans*, there are two *nox* genes, i.e., *nox-1* and *nox-2*, which encode a hydrogen peroxide-forming Nox-1 and a water-forming Nox-2, respectively [83]. However, only one Nox, either Nox-1 or Nox-2, is present in many other bacterial species. For example, *Mycoplasma bovis* possesses Nox-1 [84], whereas *Streptococcus pneumoniae*, Group B *Streptococcus*, and *Streptococcus sanguinis* possess Nox-2 [82,85,86]. In *S. suis*, a homolog of Nox-2 was identified to be regulated by the SpxA1 regulator [81]. The *nox* gene deletion mutant displayed reduced tolerance to oxidative stress induced by environmental oxygen, hydrogen peroxide, and SIN-1. Deletion of *nox* resulted in attenuated virulence in *S. suis* in both murine and pig infection models [44]. Very recently, in vivo transcriptome analysis and coinfection experiments further confirmed the involvement of Nox in *S. suis* virulence [45]. In addition, it was demonstrated that the enzymatic activity of Nox contributed significantly to oxidative stress resistance, and to a lesser extent, to the virulence of *S. suis* [44]. Given that Nox of *S. pneumoniae* elicits a protective immune response in mice, *S. suis* Nox may have vaccine potential.

## 7. Nucleases

Two nucleases, i.e., SsnA and EndAsuis, have been intensively studied in *S. suis* [46,47,48,49,87]. SsnA possesses a secretion signal peptide sequence at the N-terminus and a cell wall anchoring motif (LPKTG) at the C-terminus [46,87]. In accordance with its structure, SsnA is cell-wall located, with a portion secreted into the supernatant [47]. SsnA targets single- and double-stranded linear DNA, and its activity is dependent on Ca^2+^ and Mg^2+^ [46,87]. Reverse transcription-PCR analysis showed that the *ssnA* gene is expressed throughout the *S. suis* growth stages and western blotting revealed that SsnA is expressed during infection. Results from different research teams all demonstrated that SsnA plays a role in the pathogenesis of *S. suis*. Fontaine et al. showed that most of the *S. suis* field strains isolated from internal organs displayed a nuclease phenotype, whereas less than half of the surface isolates exhibited the same phenotype [87]. Consistently, comparative proteomics analysis revealed that SsnA is expressed in a virulent *S. suis* 9 strain, but is absent in an avirulent strain [88]. Haas et al. found that a DNase-deficient mutant, in which transposon Tn917 was inserted into the *ssnA* gene, exhibited attenuated virulence in an amoeba model and induced lower levels of cytokines and matrix metalloproteinase-9 in a macrophage model [46]. de Buhr et al. demonstrated that SsnA is involved in the degradation of human and porcine neutrophil extracellular traps (NETs), thus protecting *S. suis* against the antimicrobial activity mediated by NETs [47]. Recently, Li et al. confirmed the role of SsnA in *S. suis* virulence. The *ssnA* deletion mutant showed markedly decreased adherence to and invasion of HEp-2 cells. Deletion of *ssnA* in *S. suis* led to attenuated virulence in a CD1 mouse infection model [48]. The recombinant SsnA protein could elicit a significant immune response in mice and pigs. However, only mice were protected against *S. suis* challenge [89,90].

EndAsuis is a novel nuclease of *S. suis* that showed a high level of homology to the pneumococcal endonuclease A. EndAsuis is cell membrane-anchored, and its activity could not be detected in the supernatant. In contrast to SsnA, the activity of EndAsuis is dependent on Mg^2+^, but not on Ca^2+^. Interestingly, although EndAsuis is involved in the degradation of NETs, the *endAsuis* deletion mutant exhibited no significant difference in resistance to the antimicrobial activity mediated by neutrophils or NETs compared to the parent strain [49]. Further studies are necessary to elucidate the role of EndAsuis in the pathogenesis of *S. suis*.

## 8. Enolase

Enolase of *S. suis* has attracted a lot of attention since it was first identified. Enolase could bind to extracellular matrix components, including fibronectin, plasminogen, fibrinogen, and laminin [50,51,52,53], which further promotes *S. suis* adhesion to and invasion of host cells. Moreover, the involvement of enolase in *S. suis* adhesion to host cells has been clearly demonstrated using various methods [50,53,54,55,56]. Through interactions with human fibrinogen, enolase contributes to *S. suis* resistance to phagocytosis by neutrophils, thus enhancing *S. suis* survival in human blood [52]. In addition, *S. suis* enolase plays a role in disrupting the integrity of the blood-brain barrier by inducing interleukin-8 release [57]. Very recently, enolase of *S. suis* was identified to be a pig and human IgG-binding protein, and the two binding domains in the C-terminal exhibited specificity to interact with pig and human IgGs [58].

While there is no debate about the role of enolase in the pathogenesis of *S. suis*, the subcellular localization and vaccine potential of enolase appear to be strongly controversial. Feng et al. showed that *S. suis* enolase is a cell surface protein using multiple approaches, whereas Esgleas et al. reported that enolase is present in the supernatant, cell wall, and cytoplasm [50,56]. Recently, Liu et al. showed that enolase was significantly increased in both secreted and surface-associated fractions of the *prsA* deletion mutant [64]. Based on these results, we speculate that after synthesis in the cell, enolase can be transported to the cell surface of *S. suis*, with a portion secreted to the supernatant. It is worth noting that studies carried out by different research teams obtained opposite results regarding the protective ability of enolase against *S. suis* infection in mice, despite a strong antibody response being induced following immunization with this protein [55,56,91,92]. Considering that these studies were conducted using a mouse infection model, further studies using the natural host of *S. suis* (pig) are still required.

## 9. S-ribosylhomocysteinase (LuxS)

**Figure 1:**
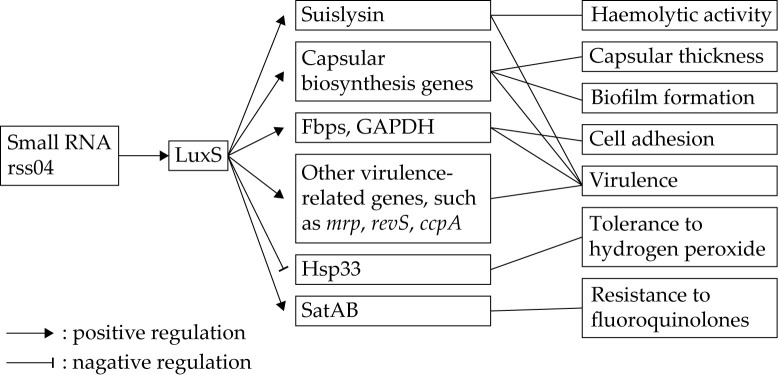
Gene regulation and functions of S-ribosylhomocysteinase (LuxS) in *S. suis*. LuxS is positively regulated by small RNA rss04. LuxS regulates the expression of multiple genes associated with various phenotypes of *S. suis*.

LuxS is one of the enzymes required for the production of autoinducer-2 (AI-2, a signal molecule involved in quorum sensing), although the transcription level of *luxS* is not correlated with AI-2 production [93,94]. In *S. suis*, expression of *luxS* is positively regulated by small RNA rss04 [95]. The functions of LuxS in the physiology and pathogenesis of *S. suis* have been extensively studied. Figure 1 shows the gene regulation and functions of LuxS in *S. suis*. Deletion of *luxS* in *S. suis* led to various phenotypic changes, including impaired growth, decreased biofilm formation and hemolytic activity, reduced adherence to epithelial cells, thinner capsular, enhanced resistance to hydrogen peroxide, and increased susceptibility to fluoroquinolones [59,60,61]. The *luxS* deletion mutant also displayed attenuated virulence in both zebrafish and piglet infection models. The contribution of LuxS to the pathogenicity of *S. suis* might be partly due to its positive regulation of several virulence-associated genes [59,60]. The involvement of *S. suis* LuxS in the resistance to fluoroquinolones is mediated by regulating the fluoroquinolone efflux pump SatAB [61].

## 10. Peptidyl Isomerase PrsA

The peptidyl isomerase PrsA is a potential substrate of the type IV-like secretion system (T4SS) in *S. suis*. Deletion of the T4SS component VirD4 resulted in significant down-regulation of PrsA in secreted proteins upon exposure to hydrogen peroxide. PrsA exhibited significant cytotoxicity to bEnd.3 cells and induced production of proinflammatory cytokines in RAW264.7 cells [62]. It has also been demonstrated that PrsA is expressed in intracellular, surface-associated and secreted proteins. The *prsA* gene is highly conserved among *S. suis* strains, and immunization with PrsA induced antibody responses in mice and conferred protection against both *S. suis* 2 and *S. suis* 9 challenges [63]. Recently, the role of PrsA in the pathogenesis of *S. suis* has been partly elucidated. Deletion of *prsA* resulted in increased chain length, decreased growth, enhanced adhesion to but weakened invasion of host epithelial cells, reduced survival in RAW264.7 cells and pig whole blood, and attenuated virulence in mice. Suilysin, a virulence factor involved in the hemolytic activity of *S. suis*, was markedly reduced in surface-associated and secreted proteins of the *prsA* gene deletion mutant. In contrast, glyceraldehyde-3-phosphate dehydrogenase (GAPDH) and enolase, two adhesion-associated factors of *S. suis*, were significantly increased [64]. These results suggested that PrsA could be involved in the secretion of selected virulence factors, thus contributing to the pathogenesis of *S. suis*.

## 11. (p)ppGpp Synthetases

The alarmones guanosine tetraphosphate and guanosine pentaphosphate, collectively termed (p)ppGpp, are involved in the regulation of growth and stress responses in bacteria. (p)ppGpp synthetases play a key role in controlling the cellular levels of (p)ppGpp [96]. There are two (p)ppGpp synthetases, i.e., RelA and RelQ, in *S. suis*. Simultaneous deletion of RelA and RelQ resulted in different phenotypes and attenuated pathogenicity compared to the wild type *S. suis* strain. The mutant exhibited a longer chain, a reduced ability to adhere to and invade HEp-2 cells, decreased resistance to blood killing and phagocytosis by THP-1 cells, attenuated virulence in mice, and ensured easier clean-up in mouse tissues. Moreover, the expression of several virulence factors was down-regulated in the mutant, suggesting that (p)ppGpp synthetases could modulate virulence genes expression in *S. suis* [65]. Zhang et al. further explored the role of individual (p)ppGpp synthetases in the stringent response induced by glucose starvation. The results showed that only RelA plays a role in the adaptation to glucose starvation. Transcriptome analysis revealed that RelA is involved in the regulation of protein synthesis, DNA replication, cell division and growth, cell wall/membrane biogenesis, carbohydrate transport, glycolysis, and carbon catabolite in *S. suis* [66]. Recently, it was shown that the CodY regulator could bind to the promoter of *relA* in a manner independent of GTP, and the expression of *relA* was positively regulated by CodY in *S. suis* [97].

## 12. Conclusions

*S. suis* remains one of the most severe swine bacterial pathogens and it is a serious threat to public health. Understanding of the physiology and pathogenesis of *S. suis* undoubtedly contributes to the control of its infections. This review highlights the role of various enzymes in the physiology and pathogenesis of *S. suis*. Many enzymes have a role, either confirmed or potential, in the physiology and pathogenesis of *S. suis*, despite only some representative enzymes being introduced here. It should be noted that previous studies were mainly carried out in *S. suis* 2. Since some enzymes are present in various serotypes of *S. suis*, further investigation of these enzymes in other serotypes of *S. suis* should be performed. For certain enzymes, the controversial results obtained from different teams need to be clarified. Given that enzymes play important roles in a wide variety of intracellular processes, a promising approach is to design novel antimicrobial drugs targeted to certain enzymes.

## Figures and Tables

**Table 1:** Characterization of some representative enzymes in *Streptococcus suis*.

Enzyme	Main Functions	References
CopA	Copper efflux	[20]
PmtA	Ferrous iron and cobalt efflux, tolerance to hydrogen peroxide-induced oxidative stress	[21]
MsmK	Utilization of multiple carbohydrates, pathogenesis	[22,23]
Ide_Ssuis_	Degradation of porcine IgM, complement evasion	[24,25,26]
IgdE	Degradation of porcine IgG	[27]
IgA1 protease/ZmpC	Degradation of human IgA1, pathogenesis ^1^	[28,29,30,31]
eSTK	Maintaining bacterial morphology, tolerance to stresses, pathogenesis	[32,33,34]
eSTP	Virulence, cell adhesion, and immune evasion ^2^	[35,36]
SspA-1	Virulence, trigger of proinflammatory cytokines	[37,38]
SspA-2	Pathogenesis, proinflammatory response in macrophages	[39,40,41]
Superoxide dismutase	Oxidative stress resistance, virulence	[42,43]
NADH oxidase	Tolerance to oxidative stress, virulence	[44,45]
SsnA	Degradation of human and porcine neutrophil extracellular traps, pathogenesis	[46,47,48]
EndAsuis	Degradation of neutrophil extracellular traps	[49]
Enolase	Binding of extracellular matrix components, pathogenesis	[50,51,52,53,54,55,56,57,58]
LuxS	Growth, biofilm formation, capsule synthesis, hydrogen peroxide resistance, resistance to fluoroquinolones, pathogenesis	[59,60,61]
Peptidyl isomerase PrsA	Induction of proinflammatory cytokines, secretion of selected virulence factors, pathogenesis	[62,63,64]
(p)ppGpp synthetases (RelA, RelQ)	Pathogenesis, adaptation to glucose starvation (RelA)	[65,66]

^1^ The role of IgA1 protease/ZmpC in the degradation of human IgA1 and in the pathogenesis of *S. suis* is controversial. ^2^ The role of eSTP in cell adhesion and immune evasion appears to be contradictory between *S. suis* 2 and 9.
